# Generation of an induced pluripotent stem cell line (TRNDi004-I) from a Niemann-Pick disease type B patient carrying a heterozygous mutation of p.L43_A44delLA in the *SMPD1* gene

**DOI:** 10.1016/j.scr.2019.101436

**Published:** 2019-04-12

**Authors:** Amanda Baskfield, Rong Li, Jeanette Beers, Jizhong Zou, Chengyu Liu, Wei Zheng

**Affiliations:** a National Center for Advancing Translational Sciences, National Institutes of Health, Bethesda, MD, USA; b iPSC core, National Heart, Lung and Blood Institute, National Institutes of Health, Bethesda, MD, USA; c Transgenic Core, National Heart, Lung and Blood Institute, National Institutes of Health, Bethesda, MD, USA

## Abstract

Niemann-Pick disease type B (NPB) is a rare autosomal recessive lysosomal storage disease caused by mutations in the *SMPD1* gene, which encodes for acid sphingomyelinase. A human induced pluripotent stem cell (iPSC) line was generated from dermal fibroblasts of a 1-year old male patient with NPB that has a heterozygous mutation of a p.L43_A44delLA of SMPD1 using non-integrating Sendai virus technique. This iPSC line offers a useful resource to study the disease pathophysiology and as a cell-based model for drug development to treat NPB.

## Resource utility

This human iPSC line is a useful tool for studies of disease phenotype and pathophysiology, and use as a cell-based disease model for drug development to treat patients with NPB.

## Resource details

Niemann-Pick disease type B (NPB) is a rare autosomal recessive, lysosomal storage disease that is caused by mutations in the *SMPD1* gene, leading to a deficiency in acid sphingomyelinase in the lysosome of patient cells. This deficiency leads to an accumulation of sphingomyelin in the lysosome, resulting in hepatosplenomegaly and other symptoms in NPB patients (Levran et al., 1991; Wasserstein and Schuchman, 1993).

**Fig. 1:**
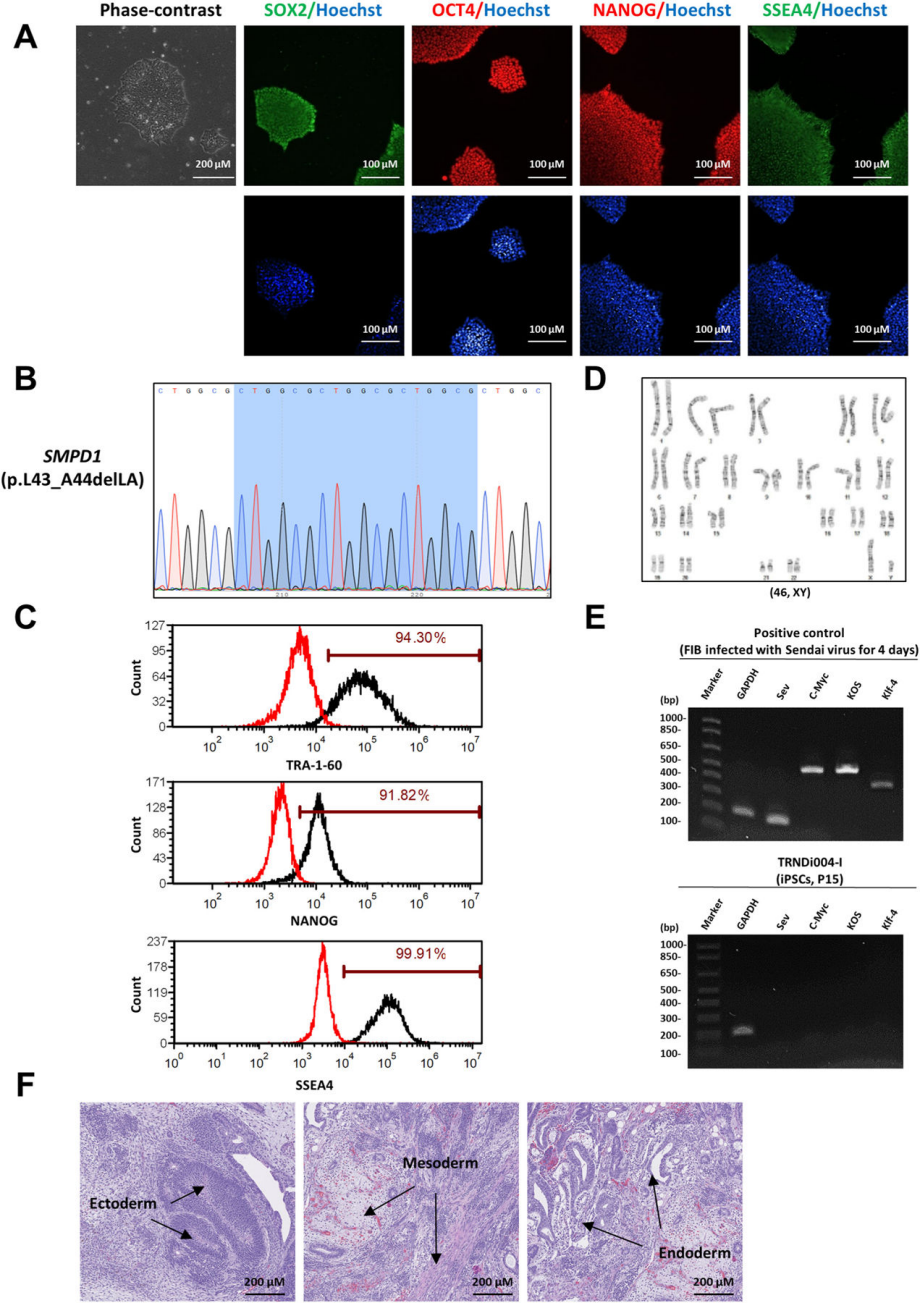
Characterization of TRNDi004-I iPSC line. **A)** Left: phase contrast imaging of TRNDi004-I colonies grown on Geltrex. Right: Representative immunofluorescent images of iPSCs positive for stem cell markers: SOX2, OCT4, NANOG, and SSEA4. Nucleus is labelled with Hoechst (blue). **B)** Detection of a heterozygous gene mutation of p.L43_A44delLA in the SMPD1 gene of iPSC TRNDi004-I. **C)** Flow cytometry analysis of pluripotency protein markers: TRA-1–60, NANOG and SSEA4. **D)** Cytogenetic analysis showing a normal karyotype (46, XY). **E)** RT-PCR verification for the clearance of the Sendai virus from reprogrammed cells. Sendai virus vector transduced fibroblasts were used as positive control. **F)** Pathological analysis of teratoma from TRNDi004-I iPSC, showing a normal ectodermal, mesodermal, and endodermal differentiation.

In this study, a human induced pluripotent stem cell (iPSC) line TRNDi004-I was established from dermal fibroblasts of a 1-year-old male patient (Table 1). The integration-free CytoTune-Sendai viral vector kit (A16517, Thermo Fisher Scientific) containing OCT 3/4, KLF4, SOX2, and c-MYC pluripotency transcription factors was used to transduce the patient fibroblasts using methods previously stated (Chen et al., 2011; Beers et al., 2015). Genetic analysis shows that this iPSC line carries a heterozygous gene mutation of p.L43_A44delLA in exon 1 of *SMPD1* (Fig. 1B). The cells exhibit a classical embryonic stem cell morphology (Fig. 1A) and carry a normal karyotype (46, XY), as confirmed by G-banding karyotype analysis (Fig. 1D). Immunohistochemistry staining and flow cytometry analysis demonstrated high expression levels of major pluripotency protein markers of NANOG, SOX2, OCT4, SSEA4, and TRA-1–60 on those cells (Fig. 1A and C). Sendai virus (SeV) clearance was verified with reverse transcription polymerase chain reaction (RT-PCR) using SeV-specific primers with no virus present by passage 15 (Fig. 1E). The iPSC line was not contaminated with mycoplasma (Supplementary Fig. S1) and was authenticated using STR DNA analysis, which demonstrated matching genotype at all 18 loci examined (information available with the authors). Furthermore, the pluripotency of this iPSC line was confirmed by the teratoma formation experiment that exhibited its ability to differentiate into cells/tissues of all three germ layers (ectoderm, mesoderm and endoderm) *in vivo* (Fig. 1F).

## Materials and methods

### Cell culture

Patient skin fibroblasts were obtained from Coriell Cell Repositories (GM11097) and cultured in DMEM (Thermo Fisher Scientific), supplemented with 10% fetal bovine serum (FBS), 100 units/ml penicillin, and 100 μg/ml streptomycin in a humidified incubator with 5% CO_2_ at 37 °C. TRNDi004-I iPSCs were cultured in StemFlex medium (Thermo Fisher Scientific) on Geltrex (Thermo Fisher Scientific)-coated plates. The cells were maintained at 5% CO_2_, 5% O_2_ at 37 °C and the cells were passaged with 0.5 mM ethylenediaminetetraacetic acid (EDTA) when colonies were approximately 70% confluent.

### Reprogramming of human skin fibroblasts

Fibroblast cells were reprogrammed into iPSCs using the integration-free CytoTune Sendai viral vector kit (A16517, Thermo Fisher Scientific) following the methods previously stated (Chen et al., 2011; Beers et al., 2015).

### Immunocytochemistry staining

For immunofluorescence staining, patient iPSCs were fixed with 4% paraformaldehyde for 30 min at RT, permeabilized with 0.3% Triton X-100 in Dulbecco’s phosphate-buffered saline (DBPS) for 15 min, and washed with DPBS. Cells were blocked using Image-iT™ FX signal enhancer (Thermo Fisher Scientific) for 1 h and incubated with primary antibodies, including SOX2, OCT4, NANOG and SSEA4, overnight at 4 °C. Cells were then washed and incubated with corresponding secondary antibody conjugated with Alexa Fluor 488 or Alexa Fluor 594 for 1 h at room temperature (antibodies used are listed in Table 2). Cells were washed and stained with Hoechst 33342 for 15 min and imaged using an INCell Analyzer 2200 imaging system (GE Healthcare) with 20× objective lens and Texas Red, FITC and DAPI filter sets.

### Genome analysis of variant in SMPD1 gene

The genome analysis of variants in the *SMPD1* gene was conducted through Applied StemCell (Milpitas, CA, USA). Genomic DNA was extracted from iPSC line TRNDi004-I followed by PCR amplification using MyTaq™ Red Mix (Bioline, Taunton, MA). Amplifications were carried out using the following program: 95 °C, 2 min; 35 cycles of [95 °C, 15 s; 60 °C, 15 s; 72 °C, elongation duration varies by amplificon size], 72 °C, 5 min; 4 °C, indefinite. PCR products were subsequently sequenced by using Sanger sequencing analysis to identify potential mutations. The specific primers for gene amplification and sequencing are listed in Table 2. The heterozygous gene mutation of p.L43_A44delLA in exon 1 of *SMPD1* was further confirmed by Codex BioSolutions, Inc. (Gaithersburg, MD).

### Flow cytometry analysis

The iPSCs were harvested using TrypLE Express enzyme (Thermo Fisher Scientific). Cells were fixed with 4% paraformaldehyde for 10 min at room temperature and then washed with DPBS. Before fluorescence-activated cell sorting analysis, cells were permeabilized with 0.2% Tween-20 in DPBS for 10 min at room temperature and stained with fluorophore-conjugated antibodies for 1 h at 4 °C on a shaker. Relative fluorophore-conjugated animal nonimmune immunoglobulin was used as the negative control (antibodies and nonimmune immunoglobulin used are listed in Table 2). Cells were then analyzed on a BD AccuriC6 Flow Cytometry system (BD Biosciences).

### G-banding karyotype

The G-banding karyotype analysis was performed at WiCell Research Institute (Madison, WI, USA). A total of 20 randomly selected metaphases were analyzed by G-banding.

### Testing for Sendai reprogramming vector clearance

Total RNA was isolated from iPSCs TRNDi004-I of passage 15 using RNeasy Plus Mini Kit (Qiagen). Human fibroblasts (Coriell Institute, GM05659), after infection with Sendai virus for 4 days, were used as a positive control. A total of 1 μg RNA/reaction was reverse transcribed with SuperScript™ III First-Strand Synthesis SuperMix kit, and PCR was performed using Platinum II Hot Start PCR Master Mix (Thermo Fischer Scientific) with the primers listed in Table 2. The products were then loaded into an *E*-Gel® 1.2% with SYBR Safe™ gel and run at 120 V electric field. Finally, the image was collected using G: Box Chemi-XX6 gel doc system from Syngene (Frederick, MD).

### Short tandem repeat (STR) DNA profile analysis

NPB patient fibroblasts and iPSCs were sent to the John Hopkins University Genetic Resources Core Facility for STR DNA profile analysis using a Promega PowerPlex 18D Kit. The PCR product was electrophoresed on an ABI Prism® 3730xl Genetic Analyzer and data were analyzed using GeneMapper® v 4.0 software (Applied Biosystems).

### Mycoplasma detection

Mycoplasma testing was performed and analyzed using the Lonza MycoAlert kit (Lonza), following the protocol from the company. (Ratio B/A > 1.2 mycoplasma positive; 0.9–1.2 results ambiguous; < 0.9 mycoplasma negative).

### Teratoma formation assay

Patient iPSCs cultured in 6- well plates were dissociated with DPBS containing 0.5 mM EDTA and approximately 1 × 10^7^ dissociated cells were collected and re-suspended in 400 μl culture medium supplied with 25 mM HEPES (pH 7.4) and stored on ice. Then, 50% volume (200 μl) of cold Matrigel (354277, Corning) was added and mixed with the cells. The mixture was injected subcutaneously into NSG mice (JAX No. 005557) at 150 μl per injection site. Visible tumors were removed 6–8 weeks post injection that were immediately fixed in 10% Neutral Buffered Formalin. The fixed tumors were embedded in paraffin and stained with hematoxylin and eosin. Images were collected and analyzed using the NanoZoomer Digital Pathology software (Hamamatsu).

## Supplementary Material

1

## Figures and Tables

**Table:** Resource table

Unique stem cell line identifier	TRNDi004-I
Alternative name(s) of stem cell line	HT222I
Institution	National Institutes of Health National Center for Advancing Translational Sciences Bethesda, Maryland, USA
Contact information of distributor	Dr. Wei Zheng Wei.Zheng@nih.gov
Type of cell line	iPSC
Origin	Human
Additional origin info	Age: 1-year-old
	Sex: Male
	Ethnicity: N/A
Cell source	Skin dermal fibroblasts
Clonality	Clonal
Method of reprogramming	Integration-free Sendai viral vectors
Genetic modification	NO
Type of modification	N/A
Associated disease	Niemann-Pick disease type B (NPB)
Gene/locus	Gene: *SMPD1*
	Locus: 11p15.4
	Mutation: p. L43_A44delLA
Method of modification	N/A
Name of transgene or resistance	N/A
Inducible/constitutive system	N/A
Date archived/stock date	2018
Cell line repository/bank	N/A
Ethical approval	NIGMS Informed Consent Form was obtained from patient at time of sample submission. Confidentiality Certificate: CC-GM-15–004

**Table 1:** Characterization and validation.

Classification	Test	Result	Data
Morphology	Photography	Normal	Fig. 1 Panel A
Phenotype	Immunocytochemistry	SOX2, OCT4, NANOG, SSEA4	Fig. 1 Panel A
	Flow cytometry	TRA-1–60 (94.30%); NANOG (91.82%); SSEA4 (99.91%)	Fig. 1 Panel C
Genotype	Karyotype (G-banding) and resolution	46XY Resolution: 500–550	Fig. 1 Panel D
Identity	Microsatellite PCR (mPCR) OR	Not performed	N/A
	STR analysis	18 sites tested, all sites matched	Available from the authors
Mutation analysis (If Applicable)	Sequencing	Heterozygous mutation of *SMPD1*, p.L43_A44delLA	Fig. 1 Panel B
	Southern Blot OR WGS	N/A	N/A
Microbiology and virology	Mycoplasma	Mycoplasma testing by luminescence. Negative	Supplementary Fig. S1
Differentiation potential	Teratoma formation	Teratoma with three germlayers formation (ectoderm, mesoderm and endoderm)	Fig. 1 Panel F
Donor screening (Optional)	HIV 1 + 2 Hepatitis B, Hepatitis C	N/A	N/A
Genotype additional info (Optional)	Blood group genotyping	N/A	N/A
	HLA tissue typing	N/A	N/A

**Table 2:** Reagents details.

Antibodies used for immunocytochemistry/flow-cytometry			

	Antibody	Dilution	Company Cat # and RRID
Pluripotency markers	Mouse anti-SOX2	1:50	R & D Systems, Cat# MAB2018, RRID: AB_358009
Pluripotency markers	Rabbit anti-NANOG	1:400	Cell Signaling Technology, Cat# 4903, RRID: AB_10559205
Pluripotency markers	Rabbit anti-OCT4	1:400	Thermo Fisher, Cat# A13998, RRID: AB_2534182
Pluripotency markers	Mouse anti-SSEA4	1:1000	Cell Signaling Technology, Cat# 4755, RRID: AB_1264259
Secondary antibodies	Donkey anti-Mouse IgG (Alexa Fluor 488)	1:400	Thermo Fisher, Cat# A21202, RRID: AB_141607
Secondary antibodies	Donkey anti-Rabbit IgG (Alexa Fluor 594)	1:400	Thermo Fisher, Cat# A21207, RRID: AB_141637
Flow cytometry antibodies	Anti-Tra-1–60-DyLight 488	1:50	Thermo Fisher, Cat# MA1–023-D488X, RRID: AB_2536700
Flow Cytometry Antibodies	Anti-Nanog-Alexa Fluor 488	1:50	Millipore, Cat# FCABS352A4, RRID: AB_10807973
Flow cytometry antibodies	anti-SSEA-4-Alexa Fluor 488	1:50	Thermo Fisher, Cat# 53–8843–41, RRID: AB_10597752
Flow cytometry Antibodies	Mouse-IgM-DyLight 488	1:50	Thermo Fisher, Cat# MA1–194-D488, RRID: AB_2536969
Flow Cytometry antibodies	Rabbit IgG-Alexa Fluor 488	1:50	Cell Signaling Technology, Cat# 4340S, RRID: AB_10694568
Flow cytometry antibodies	Mouse IgG3-FITC	1:50	Thermo Fisher, Cat# 11–4742–42, RRID: AB_2043894

Primers			

	Target	Forward/Reverse primer (5′−3′)	

Sev specific primers (RT-PCR)	Sev/181 bp	GGA TCA CTA GGT GAT ATC GAG C/ACC AGA CAA GAG TTT AAG AGA TAT GTA TC	
Sev specific primers (RT-PCR)	KOS/528 bp	ATG CAC CGC TAC GAC GTG AGC GC/ACC TTG ACA ATC CTG ATG TGG	
Sev specific primers (RT-PCR)	Klf4/410 bp	TTC CTG CAT GCC AGA GGA GCC C/AAT GTA TCG AAG GTG CTC AA	
Sev specific primers (RT-PCR)	C-Myc/523 bp	TAA CTG ACT AGC AGG CTT GTC G/TCC ACA TAC AGT CCT GGA TGA TGA TG	
House-Keeping gene (RT-PCR)	GAPDH/197 bp	GGA GCG AGA TCC CTC CAA AAT/GGC TGT TGT CAT ACT TCT CAT GG	
Targeted mutation analysis (PCR)	SMPD1–1/559 bp	GTC AGC CGA CTA CAG AGA AGG/TAG ATG CCA CCC TCT CCA TCA G	
Targeted mutation analysis (PCR)	SMPD1–2/454 bp	CTG AAG GTG AGC ACT GAA GG/TGG TGA GAA ATC AGA GGC AG	
Targeted mutation analysis (PCR)	SMPD1–3/457 bp	ATC TGG AAG GCA AAG GTG TG/GTC AGT GAG GAA GAG GAT GC	
Targeted mutation analysis (PCR)	SMPD1–4/576 bp	TGG AAC ATC TCT TTG CCT ACT GTG/CGA TAA GTA CCT GAG GGT GC	
Targeted mutation analysis (PCR)	SMPD1–5/805 bp	GCA CAC CTG TCA ATA GCT TCC CT/TGA AGG AGG GTG GCT GGA GAT	
Targeted mutation analysis (PCR)	SMPD1–6/164 bp	CCT TTC TAC TCT TAT CTC CAG CCA C/GGG AAG ATG ACA TGG GAT GG	
Targeted mutation analysis (PCR)	SMPD1–7/443 bp	ACC TTA TCC ATC CCA TGT CAT CTT CC/TCT CCT CGA TCC TCA GCA GC	
Targeted mutation analysis (PCR)	SMPD1–8/483 bp	GTC TCC GCC TCA TCT CTC TC/ACT CAC CCT GTC CCT ATT CC	
Targeted mutation analysis (PCR)	SMPD1–9/490 bp	TCC TTA ATT CTC CCT ACT AGG TGC/CCC ACC AAC TCC AGG ATA AG	
Targeted mutation analysis (PCR)	SMPD1–10/533 bp	CTG GCT GCT CAG TTC TTT GG/TGG TAG AGA AAC CAG AAG GTC	
Targeted mutation analysis (PCR)	SMPD1–11/460 bp	CAT ACC GCA CTG GCA GCT TC/CTC CAG GAA AGG AGA AGG TC	
Targeted mutation analysis (PCR)	SMPD1–12/656 bp	TGG CAG CTT CTC TAC AGG GC/TAA CAG ACT GGC AGC ATC AGG T	
