# Supplementary figures and images for: Generation of an induced pluripotent stem cell line (TRNDi004-I) from a Niemann-Pick disease type B patient carrying a heterozygous mutation of p.L43_A44delLA in the SMPD1 gene
Source: Stem Cell Res. Author manuscript; Available in PMC 2019 Jul 22. (PMC6643268; doi:10.1016/j.scr.2019.101436)

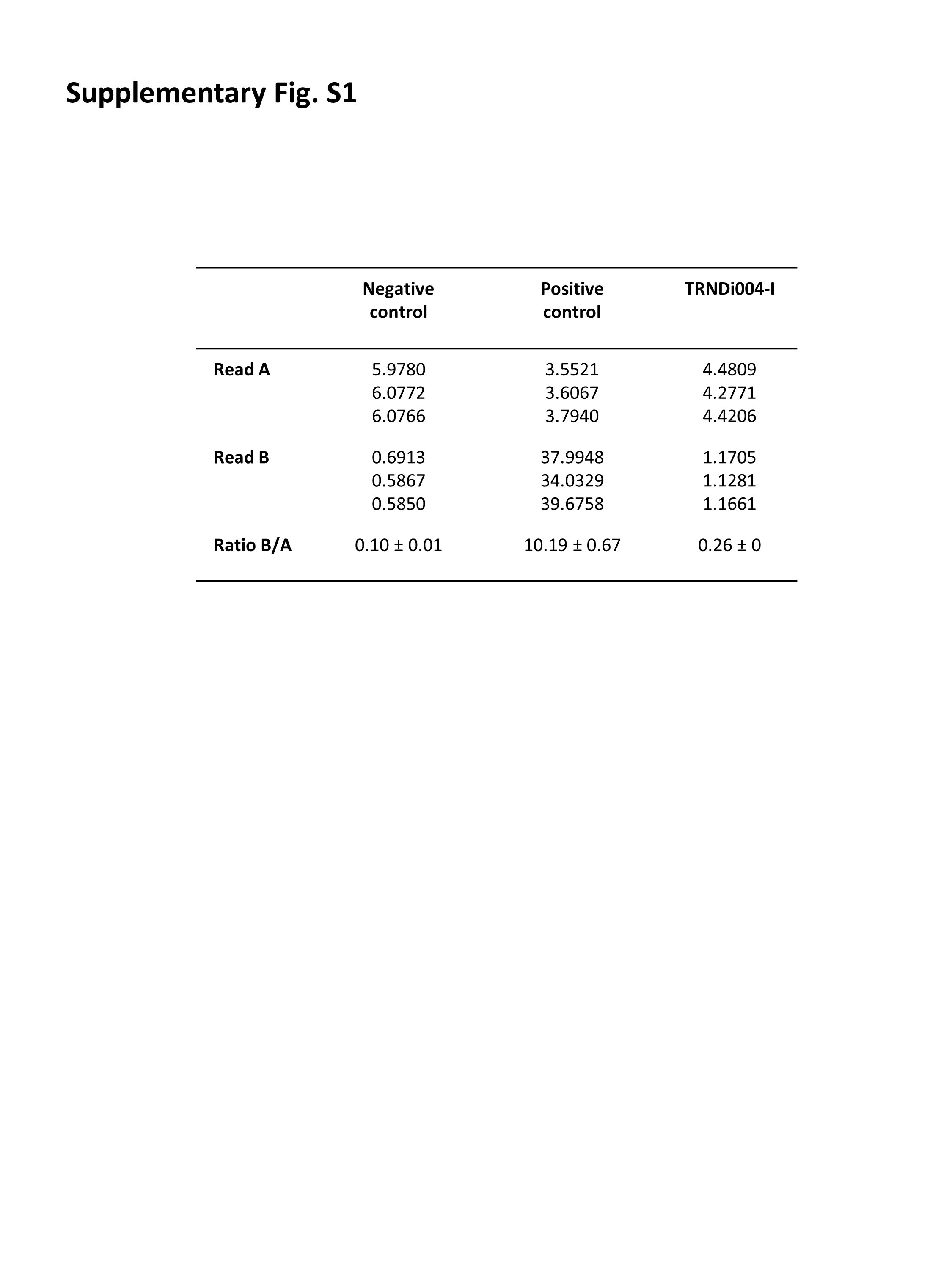

Supplement: 1 [file NIHMS1530923-supplement-1.tif]
